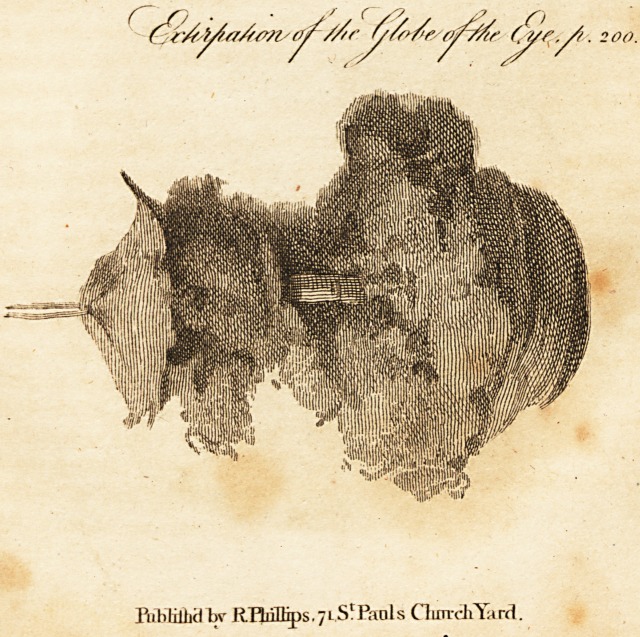# Observations on Select Subjects in Surgery

**Published:** 1803-03-01

**Authors:** W. Simmons

**Affiliations:** Manchester


### Medical and Physical Journal.

VOL. IX.] MARCH 1, 1803. [NO. XLIX.

*Printed for R. PHILLIPS, by W. Thorne, Red Lion Court, Fleet Street, London.*

### OBSERVATIONS *on* SELECT SUBJECTS *in* SURGERY; *communicated by* MR. W. SIMMONS, *of Manchester.*

### ON CONCUSSION OF THE BRAIN.

THE term *concussion* conveys not a precise idea of that derangement which is produced in the organization of the brain by external violence, on which account, and as more expressive of it, by association at least, I have been induced to substitute that of *contusion.* To this change of appellation I was led, by reflecting on the similarity of the occasional cause, as well as on the alteration of structure, and other circumstances, which mark the correspondence between an external contusion, and what has been denominated a concussion. In the former, besides the intumescence, the loss or diminution of the functions of the injured organ, effusion of blood from the ruptured vessels, producing ecchymosis or extravasation, and lastly inflammation, are the immediate and early consequences. In the latter, or *contusion* of the brain, as far as they are discernible, the effects are precisely the same, the senses are confused, there is more or less of extravasation, and a correspondent degree of inflammation. But here, as in every other accident, the event will materially depend on the importance of the organ to life; so that a blow upon the head, which might end in death, would occasion only a local pain and temporary loss of power, were its force spent upon an external muscle.

For the same reasons as above, I should call that *a slighter degree of contusion,* which has been usually called *a commotion.*

The treatment in either instance will not be varied; yet as language, when appropriate, will present the object more distinctly to the mind, the change of terms which is here proposed, will enable the surgeon to employ the means of recovery with a better chance of success, and also with more satisfaction to himself.

Mr. Pott has recorded several cases of *contusion* of the brain, wherein the patients recovered contrary to his expectation, and in a manner for which he could not account. In those cases, he had bled the patients very copiously, and used such other remedies as to him were indicated, without any advantage. When, driven to despair, and without any rational prospect of success, he bled again, and so profusely, as almost to extinguish life; by which, however, they were immediately restored to sense, and subsequently recovered. If, with deference to so great an authority, I may venture an explanation, I should now say, that the last alarming bleeding had finally subdued the inflammation, which, owing to the torpor of the brain, had become inflexible to every means short of the last measure of depletion.

The secondary symptoms consequent to such an accident, are those of inflammation; although a contrary state of the system has been said to succeed sometimes, and bear the usual signs of debility. But this I should very much doubt *a priori*, and likewise from a consideration of the soundness of the constitution preceding the violence, the nature of the occasional cause, and morbid appearances after death. In one case, where symptoms of debility were thought to prevail, and the treatment was conducted accordingly, the upper surface of the brain was found incrusted with coagulable lymph, beside other vestiges of inflammation, which were discovered in different parts of the substance of it.

Where the brain has been deeply injured, the admission of light often annoys the patient considerably; yet instances do occur both of extravasation, and of simple inflammation, where the pupils remain contracted and insensible. In what the difference consists, unless in the degree of torpor, I am unable to say. But, in this way, I should be disposed also to account for that variety in the symptoms of phrenitis, as it originates in external violence, or other occasional cause. However, the intolerance of light will furnish no criterion between *contusion* and *fracture*.

### On a Case of Cataract.

It has been deemed by some a valid objection to the operation of couching or depressing the cataract, that, should the humour re-ascend, the opacity of its capsule may may disappoint the expectations, and frustrate success. Not long since, I hit upon a method of obviating this objection; at least, in that instance it was attended with complete success. The subject of it was a poor boy, then in his nineteenth year, who had been blind from early infancy. The cataract in the left eye was soft, and depressed without any difficulty, but the anterior portion of the capsule was opaque. I therefore made several attempts to detach it from its adhesions, but failed. At the moment, it occurred to me to pass the point of the needle through the centre of the pupil into the anterior chamber of the aqueous humour, and divide the capsule horizontally. This was executed without wounding the iris; and, instantly, the severed edges receded from each other, so as to expose nearly the whole circle of the pupil. During his stay in the Infirmary they continued to recede; and, at the time of his discharge, the border only of each was discernible.

As this poor boy had no knowledge of objects by sight, I presented several to him on the third day, at the first exposure of the eye after the operation. He could readily distinguish the motion of my fingers, and said the points were upwards, which was so. But when I shewed him my hat, he was at a loss; and resorted to the touch before he could say what it was. He called a table by its name, at the distance of a yard and a half from him; but whether he had a previous knowledge of it I could not learn. When questioned as to his knowledge of colours before the operation, he replied, that he could distinguish scarlet or any glorious colour. A reciprocation in the effect of objects on sound and sight, must depend altogether on the strength of the impulse on the respective senses; consequently none, except colours which are brilliant or glaring, can produce a distinguishable perception.

The fashionable mode of operating for the cataract, at the present time, is by extraction; yet, under the authority of that excellent guide, Mr. Pott, I have hitherto confined myself to that of depression. And, if I may be permitted to give an opinion upon the subject from a moderate share of practice, I see no reason to relinquish it. It is as generally successful as any operation can be expected to be; and in simplicity there is hardly a comparison. On which account, an attempt has been made lately to render the operation by extraction more feasible, and in some measure to supply the want of adroitness in the operator. In this light, the instrument lately published by Sir

James Earle is entitled to commendation, although there are several objections to it, as from its complicated structure it must be apt to be out of order, and it can never extract a firm cataract entire. And we yet know of no signs by which to distinguish with precision whether the cataract be hard or soft, or of an intermediate degree of consistency. But, if the object of the advocates for extraction may be attained by bringing the cataract before the iris, why divide so large a portion of the transparent cornea? If firm and resisting, the needle will readily push the opaque lens through the pupil into the anterior chamber of the aqueous humour, where it will dissolve; and, if soft or fluid, multiplied experience has shewn, that it will disappear by puncturing the capsule, or lacerating the substance of the lens. As an instrument of depression, I must likewise observe, the forceps are much too unhandy ever to come into general use.

### Extirpation of the Globe of the Eye.

[ With an Engraving. ]

Fortunately this is an operation that seldom occurs; it is to the patient fraught with danger; and is more replete with horror to the surgeon than any other I ever performed. My own practice has furnished a solitary instance of it, in which my anxiety was agreeably alleviated by success. To proceed to the case:

Ann Cotton, a young woman, about twenty-three years of age, was admitted at the Infirmary, under my care, in the month of July, 1802. She came from Macclesfield, in Cheshire, where she had been employed in a branch of trade that did not exact much bodily exertion, nor particularly strain her sight. She was of a fair complexion and a healthy countenance, and, till the period of the attack, had always enjoyed good health.

When I first saw her, the globe of the left eye was protruded without the socket, and the sight of it lost. The account she gave was, that five years before she was suddenly seized, one morning at breakfast, with a severe pain over the orbit, which abated towards afternoon, but recurred again with severity in the evening, so that she got very little rest. Next day the pain intermitted, and ever afterwards recurred at uncertain periods. She was first sensible of the protrusion of the globe about four months after the attack, and at the end of two years and a half the eye became dark. Many different remedies were tried by

Mr. Simmons's Case of Lithotomy, p. 204.

Fig. 1.

Fig. 2.

Extirpation of the Globe of the Eye, p. 200.

Published by R. Phillips, 71, St. Pauls Church Yard.

by several respectable surgeons whom she consulted, without any permanent relief; and, wearied out with fruitless endeavours to obtain it, she gave up her case as hopeless at the end of three years.

The cause of the protrusion was evidently seated at the bottom of the socket, but of what nature it was could not be ascertained by a common examination; therefore an incision was made into the globe, large enough to admit the end of a finger, and the contents of it were evacuated. Only vitreous humour was discharged, the aqueous and crystalline having disappeared. I mention this circumstance particularly, because the intersections within the eye were destroyed, so that it formed one common cavity; and as it would appear, by being displaced, the crystalline humour had dissolved. By the liberty given, the tumour pushed more forward, but still the nature of it was inexplicable. Accordingly, after the inflammation produced by this examination had subsided, and a reasonable time had elapsed to see what changes might ensue, it was agreed to make a more particular inquiry. In order to this, I dissected cautiously, with the point of a lancet, until I had exposed the tumour sufficiently to feel an obscure fluctuation in it. A puncture was then made, and a small quantity only of a serous fluid discharged, the rest of the contents being of too firm a consistence to be thus evacuated. Excision of the whole now offered the only alternative, which I finished without delay with a small common scalpel. She fainted away during the operation, though she did not lose much blood, and not more than four or five ounces afterwards. This bleeding from the wound was no doubt of use in abating the subsequent inflammation, which was not violent, and submitted to one general and one topical bleeding. Nor was the danger ever thought to be imminent; and, after the second day, the symptoms gave little uneasiness. As she was at no time delirious, as the pain was confined to the socket and parts contiguous, and did not shoot back towards the hind head, I conclude that the inflammation never passed the boundary of that cavity.

The eye and tumour, which appeared to consist of a condensed scrophulous substance enclosed in a proper tunic, were removed on the 12th of August; and she was discharged cured on the 11th of October following.

I have procured a drawing to be made by an ingenious artist, of the natural size; in the upper part of which, next the thread by which it is suspended, a part of the anterior portion of the tunic is seen spread out, well depicted; lower down, the optic nerve tied with a thread; and, at the bottom, the eye itself shrunk, and much altered in its appearance. The other parts will be distinctly understood without any further explanation.

### On supposed Dislocations.

I have long entertained an opinion, that it would be advantageous to extend the limb, after the dislocation or severe contusion of a joint, where it does not, within a seasonable time, become again subservient to the purposes of volition. The secondary consequence, as in every other contusion, is inflammation, which seldom advances so high as to terminate otherwise than by resolution. But, during the inflammatory stage, membranous adhesions may and probably do form within the joint, or exterior to the capsular ligament, or both; or the muscles and tendons passing over the joint, or near it, become fixed by what has been called the adhesive inflammation; any of which impediments would hinder, and several of them combined, effectually prevent the recovery of the motion. This disability is pronounced by the people, called bone-setters, to be still a dislocation, although, in many such instances, there can be no doubt the parts had been very properly reduced. Be that as it may, they all uniformly resort to extension; and, it must be confessed, with a success which has often put even experienced surgeons to the blush.—Shall we then ascribe their better success to superior skill? That I think may be safely answered in the negative; and yet the instances are too numerous to be entirely accidental. How then shall we account for it? By making a full extension, the newly formed membranes are ruptured, and the whole is at once set free. Precisely the same effect is produced by artificial motion, and by frictions, which are the means usually employed by the regular practitioner; and these, if duly persisted in, are in general adequate to the removal of the less obstinate cause.

Finally, I have several times put it to the test of experiment; the first instance of which I shall now have permission to relate.

A man who had dislocated his shoulder, and had had it reduced by a celebrated bone-setter, came under my care at the Infirmary, as an out-patient, for the very disability which I have described. The accident had happened six weeks before, consequently it had arrived at the very period when the regular surgeon is often deserted, and for the the very same reason. This, therefore, was a case in point, and accordingly I made the necessary extension until the joint gave a crack; immediately the poor fellow expressed himself relieved, and could perform the usual motions. The relief was permanent.

Notwithstanding this account of success, I ought not to conceal the very terrible consequences which I have known to ensue from the misapplication of force to joints distorted by scrofula or other enlargement. Owing to this gross and unpardonable ignorance, I have been obliged to amputate many a limb, which might probably have been saved by timely and judicious treatment. An error so fatal is not likely to be committed by the experienced surgeon, who will know how to avail himself of the above suggestion, and also to distinguish between cases which bear even a near affinity.

### On Musk in Mortification.

My former communication on this subject (Vol. ii. p. 12) related to the history of the discovery of the efficacy of musk and volatile alkali in gangrene and mortification. That account I received from the late Dr. Darbey himself. In my own practice, the very general success of the bark in such cases, had left me without any experience of the present remedy till within a few months past. I had then occasion to try it, and with so much success that I feel it a duty to record the case.

The subject of it was a gentleman advanced in years, whose health had been for some time upon the decline. The mortification appeared upon his right leg, and continued its ravages up the limb, in defiance of every means to check it, which Mr. Ogden, of Ashton, and myself could devise. He had taken opium in large doses; wine, brandy, volatile alkali, and æther, in full but regulated quantities, besides the bark in substance to the quantity of an ounce a day, along with decoction and tincture. Nor were external applications disregarded; but all were unavailing, and not otherwise useful than by imposing a temporary check to its progress. In this forlorn hope we had recourse to musk, which he took three times a day, in the form of bolus, in doses of ten grains each, with an equal proportion of the volatile salt. From this he derived speedy and permanent relief; the mortification ceased, the dead parts exfoliated, and in no long time he perfectly recovered.

The yellow bark has been lately substituted for the pale in several diseases; but, for surgical purposes, it is generally inferior in efficacy, and particularly so in erysipelas, gangrene, and sphacelus.

### ON A CASE OF LITHOTOMY.

[ With an Engraving. ]

I cut a boy for the stone, at the Infirmary, on the 25th of June, 1802; he was about fourteen years of age, and of a stout constitution. Nothing particular occurred during the operation, which was over in a few minutes, and he recovered without any accident. But, after the operation, the stone was found to consist of two parts, which were held in contact by a common pin, which had served as a nucleus to them. The whole was not large, as both of the concretions weighed only three drachms, though that on the pointed extremity was a good deal larger than the other, and but little was detached from the surface of either in the extraction. His general health was good till within nine months of the operation, when he perceived the first symptoms of the stone. Afterwards they became exceedingly distressing, and were more urgent than in any case I had before seen. At two years of age he was seized with a dysury that lasted for near a month, but to what cause to attribute it his friends were utterly at a loss. This illness however is the only one he ever underwent, before that affliction which terminated as above.

I should suppose that no question could arise except as to the time of his swallowing the pin, for there appears no other rational way of accounting for its passing into the bladder. If we suppose that the pin was the cause of the dysury at two years of age, the symptoms of that complaint must have arisen from consent between the bladder and rectum, while penetrating through the coats of the latter, on which they were excited. After it had passed through the muscular coat of the rectum, it had lain quiescent for many years in the cellular structure between it and the bladder; at length it was disturbed, and pushed by the point foremost into the cavity of the bladder. As soon as its point had pierced through the coats of that viscus, the concretion upon its point would begin to form; and when the whole had fallen into the cavity, that upon the head. This is my own opinion, and to my mind accounts satifactorily for the difference in the size of the two concretions, which, had the operation been delayed a while longer, longer, would, by additional strata, have been converted into one solid stone. The ulcerative process by which the pin penetrated into the bladder, was no doubt slow, or the symptoms would have been much more acute in their commencement than they were noted to be.

I have thought it worth while to record this singular occurrence, and to illustrate it by a plate; though a mere inspection will satisfy the reader, as one figure represents the stone entire, and the other the two parts separate.

*Jan.* 1803.

## Figures and Tables

**Fig.1. Fig. 2. f1:**
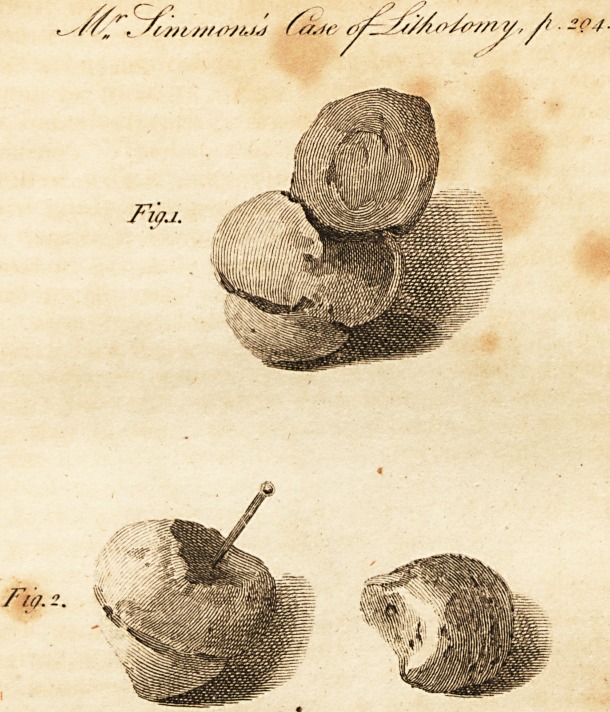


**Figure f2:**